# A Cost Decision Model Supporting Treatment Strategy Selection in BRCA1/2 Mutation Carriers in Breast Cancer

**DOI:** 10.3390/jpm11090847

**Published:** 2021-08-27

**Authors:** Nunzia Carbonara, Daniele La Forgia, Roberta Pellegrino, Cosmo Ressa, Stefania Tommasi

**Affiliations:** 1 Departments of Mechanics Mathematics and Management, Politecnico di Bari, 70126 Bari, Italy; nunzia.carbonara@poliba.it; 2 SSD Radiodiagnostica Senologica, I.R.C.C.S. Istituto Tumori “Giovanni Paolo II” di Bari, 70124 Bari, Italy; d.laforgia@oncologico.bari.it; 3 S.C. Chirurgia Plastica e Ricostruttiva, I.R.C.C.S. Istituto Tumori “Giovanni Paolo II” di Bari, 70124 Bari, Italy; m.ressa@oncologico.bari.it; 4 SSD Diagnostica Molecolare e Farmacogenetica, I.R.C.C.S. Istituto Tumori “Giovanni Paolo II” di Bari, 70124 Bari, Italy; s.tommasi@oncologico.bari.it

**Keywords:** breast cancer, BRCA-carriers management, cost decision model, economic evaluation, uncertainty

## Abstract

In this paper, a cost decision-making model that compares the healthcare costs for diverse treatment strategies is built for BRCA-mutated women with breast cancer. Moreover, this model calculates the cancer treatment costs that could potentially be prevented, if the treatment strategy with the lowest total cost, along the entire lifetime of the patient, is chosen for high-risk women with breast cancer. The benchmark of the healthcare costs for diverse treatment strategies is selected in the presence of uncertainty, i.e., considering, throughout the lifetime of the patient, the risks and complications that may arise in each strategy and, therefore, the costs associated with the management of such events. Our results reveal a clear economic advantage of adopting the cost decision-making model for benchmarking the healthcare costs for various treatment strategies for BRCA-mutated women with breast cancer. The cost savings were higher when all breast cancer patients underwent counseling and genetic testing before deciding on any diagnostic-therapeutic path, with a probability of obtaining savings of over 75%.

## 1. Introduction

Over the last decades, a number of diagnostic procedures, treatments, and follow-up programs for patients affected by breast cancer have been introduced, significantly improving patient outcomes [1]. Standard primary surgical treatment options for women with breast cancer include quadrantectomy with sentinel lymph node or with axillary dissection or mastectomy. Each surgical treatment requires different post-surgery therapies, radiotherapy, chemotherapy, and hormone therapy [2], with different long- and short-term follow-ups, such as clinical examination, mammography, ultrasound, and, in case of worsening, magnetic resonance. These different treatment options depend on breast size as well as demographic and oncological preoperative data. Breast-preserving surgery (sentinel lymph node quadrantectomy or axillary dissection and radiotherapy) is often the treatment of choice in small and early breast cancer [3].

However, in high-risk women, the possibility of the recurrence of tumor in the same breast is still high with breast-conserving therapy, as is local recurrences after mastectomy. This is the case for women with breast cancer that also have the BRCA1 or BRCA2 mutation. In the general population, the cumulative lifetime risk for breast cancer is 8–10%. In contrast, women with a BRCA mutation have a 40–80% lifetime breast cancer risk [4]. In particular, BRCA1 and BRCA2 mutation carriers have a 60–65% and 45–55% risk of breast cancer up to the age of 70 years, respectively [5,6]. In addition, mutation carriers have an increased risk of developing contralateral breast cancer and relapses after the initial breast cancer treatment [7]. Pathogenic mutations in the BRCA1 and BRCA2 genes confer high risks of breast, ovarian, and contralateral breast cancer (CBC) [6]. However, the precise magnitude of these risks is uncertain.

Risk-reducing strategies comprise intensified surveillance, lifestyle factors, chemoprevention, and risk-reducing surgeries [8].

Surveillance of BRCA1/2 mutation carriers consists of annual screening with clinical examination, mammography, and contrast-enhanced breast magnetic resonance imaging (MRI) [9,10], which detects the disease at an earlier stage [11,12]. Although breast MRI screening is highly sensitive, it has an increased rate of false positive test results and it has not been shown to reduce breast cancer mortality [13].

Beyond intensified surveillance, in high-risk women such as those that have modified BRCA genes, there are also risk-reducing surgical options, which include salpingo-oophorectomy (RRSO), bilateral mastectomy (BRRM), and contralateral mastectomy (CRRM) in women already diagnosed with BC. The incidence of BC in healthy BRCA mutation carriers can be reduced by approximately 90% through BRRM.

Despite these multiple possibilities for patients with BC and BRCA mutations, none of the guidelines proposes a single treatment path, and the different resources (drugs, radiotherapy, surgery, diagnostics, etc.) are currently used without an economic rationale [14].

The costs of the discussed screening strategies for high-risk women already diagnosed with BC are diverse, and depend on the risk profile of the patient during their lifetime [15].

For instance, in the case of breast surgery conservation with a follow-up, breast MRI screening is at least 10 times more expensive than mammographic screening and has higher diagnostic costs [16,17,18].

Substantial long-term costs are incurred with routine surveillance of the contralateral breast. These costs include but are not limited to regular physician visits, imaging studies, possible diagnostic biopsies, and eventually the inherent possibility of developing and having to treat contralateral breast cancer (CBC), which introduces additional associated costs (surgery, radiation, chemotherapy, etc.) and a psychological burden. Conversely, the costs involved with contralateral prophylactic mastectomy (CPM) as a risk-reduction strategy for CBC are related to the costly procedure and its associated short- and long-term morbidity, along with follow-up physician visits. However, no long-term intensive imaging surveillance is required and the risk of developing CBC is substantially decreased. With risk-reduction surgery, there still is a risk of complications: Bleeding, infection, chronic pain, and the need for revisions. The rates and types of complications are influenced by the choice of reconstruction and ranges from 30–64% [19]. Women undergoing implant reconstruction also need to consider the rare risk of implant-associated lymphoma and the need to have implants replaced over time [20,21,22,23].

Therefore, in the absence of optimal treatment and unique clinical recommendation for patients with BRCA-mutated breast cancer, and for the management of breast cancer risk in this population of women [14], cost may be a critical issue for the evaluation and selection of treatment options. Such a cost assessment must be performed considering the uncertainty affecting each therapeutic choice, namely considering the risks and complications that may arise with each strategy during the entire lifetime of the patient [24] and the subsequent costs required for managing such events [25].

Public health policies must combine information about the effectiveness of diverse treatment approaches to breast cancer with the economic burden associated with each strategy [26,27]. Health–economic analyses can help evaluate the monetary value of medical procedures and enable their comparison.

In this paper, a cost decision-making model is built that compares the healthcare costs for diverse treatment strategies for BRCA-mutated women with breast cancer and calculates the cancer treatment costs that could potentially be prevented if the treatment strategy with the lowest total cost along the entire lifetime of the patient is chosen. The benchmark of the healthcare costs for diverse treatment strategies for treating BRCA-mutated women with breast cancer is made in the presence of uncertainty, namely considering, throughout lifetime of the patient, the risks and complications that may arise in each strategy and, therefore, the costs associated with the management of such events.

## 2. Methods

### 2.1. Reference Case

For decision analysis, we defined a base case as a female patient who receives the first diagnosis of BC at the age 40 years, being eligible for BRCA genetic testing program, i.e., genetic counseling and testing.

We analyzed the different pathways for a BRC-mutated patient affected by breast cancer, namely undergoing breast-preserving surgery (quadrantectomy) or a unilateral mastectomy with intensive breast screening (intensive follow-up) using annual mammography and breast magnetic resonance imaging (MRI) or a contralateral and bilateral prophylactic mastectomy (risk-reducing surgery) with a subsequent ultrasound follow-up over a period of 35 or up to 75 years [28]. These indicators were selected based on the recommended starting and ending age for screening tests in the population [10,29,30]. Patients who underwent BRCA testing but did not have a BRCA1/2 mutation were not entered in the model.

The study was conducted in the multidisciplinary team of hereditary-familial tumors of the Istituto Tumori “Giovanni Paolo II”, located in Bari (Italy).

### 2.2. The Decision Analysis Model

We developed a decision model that, starting from the input data, such as the patient age, analyzes and computes the cost of each possible treatment strategy throughout the lifetime of the patient. The total cost of each therapeutic path is estimated by accounting for all the possible risky events that may occur in each specific treatment strategy. The model considers a female patient who receives the first diagnosis of BC at the age 40 years and is eligible for the BRCA genetic testing program. It adopts a 35-year time horizon; an Italian healthcare system perspective is used; and an annual discount rate of 3% is applied to the costs [4,31,32].

Considering an affected patient, the first decision is whether or not to implement a BRCA genetic testing program. This decision is based on the NCCN international guidelines combined with those provided by the Italian regional health systems [33].

Once this decision is made, different surgical options may be considered on the basis of:

- Logistical issues: Availability of genetic cancer risk assessment consultative services, adequate time to complete the BRCA genetic testing process (initial counseling, informed consent, obtaining a blood sample, 2–4 weeks for genotyping, disclosure counseling, and genetic-risk-tailored treatment advice), and the timing of the referral with respect to the current diagnostic and therapeutic treatment sequence [34];
- Clinical issues: Tumor size, number of positive axillary lymph nodes, histologic grade, and lymphovascular invasion [2], as well as the patients’ surgical choice.

In particular, for women with newly diagnosed breast cancer, the surgeon has different treatment options: Quadrantectomy without waiting for the BRCA test results, or waiting for the test and, according to the results, treat the patient with a breast resection in oncoplastic surgery and an intensified follow-up for the contralateral breast or a bilateral prophylactic mastectomy with ultrasound follow-up [31,35].

When selecting the quadrantectomy option, if the patient is positive for BRCA mutations, she may opt for intensified surveillance (clinical examination, mammography, and MRI, annually) or for prophylactic bilateral mastectomy and subsequent ultrasound follow-up. In the first case, the model estimates the cost of this path by considering that the patient may develop and have to treat contralateral breast cancer (CBC) and/or a relapse. Conversely, the prophylactic bilateral mastectomy reduces either the risk of relapse or the risk of CBC. It is a more costly procedure, there still is the risk of complications in about 10–20% of cases (bleeding, infection, chronic pain, and the need for revisions) [36,37], and the need to have implants replaced over time (about every 10–15 years) [38]. Additionally, there is a residual risk of onset of carcinoma in residual or ectopic glandular tissue in 5% of cases, which would lead to a new surgery [39].

**Figure 1:**
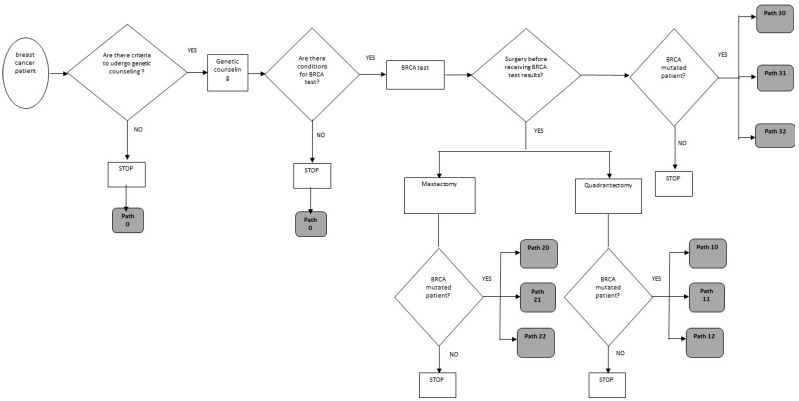
Model schematic/flow chart (a detailed description of the therapeutic paths is reported in the Appendix A).

**Figure A1:**
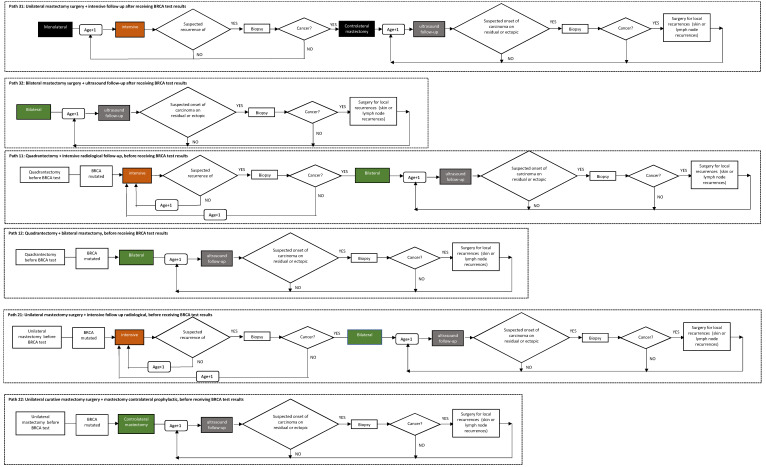
Description of therapeutic paths for patients with BRCA-mutated breast cancer.

To summarize, the possible therapeutic paths that the patient would follow without the optimization model, defined on the basis of historical data, as depicted in the flowchart in Figure 1 and detailed in the Appendix A Figure A1, are the following (herein, identified by a number):

- Path 0: Patient not subjected to genetic counseling and/or BRCA test;
- Path 10: Quadrantectomy surgery without preference for chances (quadrantectomy + intensive follow-up or quadrantectomy + bilateral mastectomy) before receiving BRCA test results;
- Path 11: Quadrantectomy + intensive radiological follow-up, before receiving BRCA test results;
- Path 12: Quadrantectomy + bilateral mastectomy, before receiving BRCA test results;
- Path 20: Mastectomy surgery with no preference for chances (unilateral mastectomy + intensive follow-up or unilateral mastectomy + contralateral prophylactic), before receiving BRCA test results;
- Path 21: Unilateral curative mastectomy surgery + intensive radiological follow-up, before receiving BRCA test results;
- Path 22: Unilateral curative mastectomy surgery + mastectomy contralateral prophylactic, before receiving BRCA test results;
- Path 30: Surgery without preference for therapeutic chance (mastectomy unilateral + radiological follow-up or bilateral mastectomy) after receiving BRCA test results;
- Path 31: Unilateral curative mastectomy surgery + intensive follow-up after receiving BRCA test results;
- Path 32: Bilateral mastectomy surgery + ultrasound follow-up after receiving BRCA test results.

The logic of the decision-making model involves comparing the costs of the different therapeutic possibilities in the presence of uncertainty and choosing the optimal one (namely, the one characterized by the lowest cost) according to the risk profile of the patient (herein, called “as is”). This is assessed on the basis of the information on the probabilities for each health state associated with clinical choices, as well as costs associated with therapeutic paths (see the Input Data subsection).

The logic of the decision-making model may be summarized in the following steps:

1. Calculation of the costs associated with the therapeutic possibilities;
2. Comparison of the costs of alternative therapeutic possibilities and the choice of the one with the lowest cost (“optimal therapeutic path”);
3. Calculation of the cost of the therapeutic path in the “as is” scenario: The therapeutic path that the patient would follow without the optimization model, defined on the basis of historical data;
4. Comparison of the cost of the “optimal therapeutic path” with the cost of the “as is” therapeutic path;
5. Calculation of the unit savings per affected patient: The cost savings that would be obtained by choosing the optimal therapeutic path, throughout the patient’s entire residual life (over a time horizon of 35 years). It is calculated by considering all the net potential savings (or costs) generated by the optimal path in each year, until the end of the life of the patient, discounted with a predefined discount rate. Specifically, the net present value (NPV) is used to calculate the present value (actual unit of savings per affected patient) of a series of future payments (with a discount rate of 3%) [31].

In order to consider the uncertainties that characterize the input data, the Monte Carlo simulation was used. It is a powerful numerical method that can consider multiple sources of uncertainty in the evaluation and decision problems, as they are in the real world [40,41]. For this reason, it is widely used to investigate costs and benefits of health strategies under uncertain conditions [4,42,43,44]. 

Therefore, rather than a single deterministic value obtained with traditional techniques, the Monte Carlo simulation provides a more realistic probabilistic representation of the model output that can be used, together with other considerations (also qualitative), to estimate the costs associated with the various therapeutic pathways during the patient’s entire useful life in the presence of uncertainty and, therefore, support decision-making. To account for uncertainty, all model parameters were varied simultaneously across their distributions, defined in the next subsections, using 10,000 simulations in a probabilistic analysis.

The simulation model was implemented using @Risk, one of the leading applications based on spreadsheets for predictive modeling, forecasting, simulation, and optimization. The developed model is easily replicable in any other simulation environment.

### 2.3. Input Data

#### 2.3.1. Probabilities

The probabilities for each health state associated with clinical choices were obtained from a comprehensive literature review. We cross-referenced these data with other past literature reviews to establish a consistency of probabilities for each health state (Table 1).

The probability of each therapeutic choice in the “as is” scenario (namely, the probability for each therapeutic path followed by the patient without the optimization model) was defined on the basis of historical data from the clinical practice in the Institute (collected by the hospital in the last years) and experts’ opinions (Table 2).

#### 2.3.2. Costs

Cost inputs are presented in Table 3. Most of the cost data used in the model are Italy-specific. In particular, due to the lack of healthcare analytical cost data in Italy, we estimated most of the costs using the Italian DRG tariffs as a proxy, which are fixed reimbursements provided by the National Health Service (NHS) (Servizio Sanitario Nazionale (SSN, Italy)) to the care providers associated with each diagnosis-related group (DRG). Although the DRG tariffs do not represent real costs to the hospital, they represent the value that the care provider charges the SSN for payment of services and are supposed to cover most hospital costs, including administration costs and overhead [50,51,52,53].

### 2.4. Sensitivity and Scenario Analysis

To evaluate the effect of input variations on the outcomes (optimal therapeutic path and unit savings per affected patient), we performed a sensitivity analysis and scenario analyses where some input parameters were varied.

We defined a plan of experiments where some main scenarios were considered, reflecting three main circumstances that answer the following questions:

- Scenario 1: What would happen if genetic counseling were extended to all patients?
- Scenario 2: What would happen if genetic counseling and BRCA testing were extended to all patients? What benefits would be gained by extending genetic counseling and testing BRCA to patients with different starting ages?
- Scenario 3: What would happen if all affected patients were treated after having received the BRCA test result? If performing the surgery after the result of the BRCA test, which therapeutic path is preferable to choose between intensive follow-up and prophylactic surgery?

## 3. Results

### 3.1. Results of Base-Case Analysis

**Figure 2:**
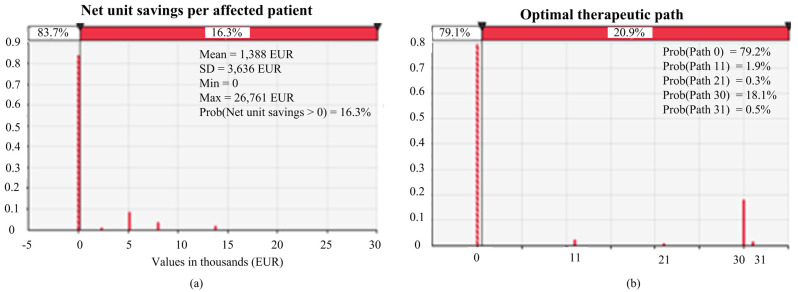
Net unit savings per affected patient (**a**) and optimal therapeutic path with the adoption of the cost decision-making model (**b**).

For our reference scenario, called the baseline model, we found that in 16% of the cases, the net unit savings per affected patient that would be obtained by choosing the optimal therapeutic path (at a lower cost) for treating mutated-BRCA patients range between EUR 0 and 26,761 with an average value of EUR 1388. In the remaining probability share, the diagnostic and therapeutic pathways chosen with the decision support system minimizing costs would not show an overall economic advantage. This first outcome is summarized in Figure 2a.

Then, we searched for the optimal therapeutic path, which is the one with the lowest cost among all possible alternative therapeutic paths.

The results of the optimal therapeutic path revealed that, excluding cases in which the patient does not undertake genetic counseling or testing (according to the input data, this situation occurred in 79% of cases), the optimal path is to intervene only after having received the outcome of the BRCA test, despite there being no preference among the therapeutic possibilities associated with this choice, in economic terms. These results are summarized in Figure 2b, which show the best performance being for therapeutic Path 30.

### 3.2. Results of Sensitivity and Scenario Analysis

The results of the sensitivity analysis show an increase in the net unit savings per affected patient in the event that counseling genetics are extended to all affected patients (Scenario 1). As summarized in Table 4 (Scenario 1 (A)), the probability of positive net unit savings per patient using the decision-making tool would rise to 34.2% of cases with an average value of EUR 2,982 up to 36,398. In this scenario, surgical intervention after receiving the BRCA test results is the optimal therapeutic path (Path 30).

If all patients undertake a BRCA genetic testing program, i.e., genetic counseling and genetic testing (Scenario 2), the savings would further expand, being positive with a probability of 75.7% with an average of EUR 6,360 per patient and could reach up to EUR 39,000, as shown in Table 2 (Scenario 2 (B)). Even in this scenario, path 30 would be the most advantageous.

Investigating the scenario of extending genetic counseling and the BRCA test to all patients with different starting ages, we obtained the net unit savings per patient with the statistics reported in Table 4 (Scenario 2 (C)) (for each age group).

Finally, assuming surgery is performed only after having received the BRCA test results (Scenario 3), we analyzed and compared the costs throughout the life of the patient with the most probable “as is” scenarios in BRCA-mutated patients, which are unilateral mastectomy with subsequent intensive radiological follow-up and bilateral mastectomy with subsequent ultrasound follow-up. The results reveal a lower total unit cost per patient with the first path compared to the second (average total cost per patient of EUR 3,034 for unilateral mastectomy with subsequent intensive radiological follow-up versus EUR 4,718 for bilateral mastectomy with subsequent ultrasound follow-up), as shown in Table 4 (Scenario 3 (D)).

## 4. Discussion and Conclusions

In this paper, we built a cost decision-making model that compares the healthcare costs for different treatment strategies for BRCA-mutated women with BC and calculates the costs of cancer treatment that could potentially be avoided if the treatment strategy with the lowest total cost along the entire lifetime of the patient is chosen for treating high-risk women with breast cancer. The assessment is performed in the presence of uncertainty, namely considering, throughout the lifetime of the patient, the risks and complications that may arise with each strategy and, therefore, the costs associated with the management of such events.

Our results reveal a clear economic advantage of adopting the cost decision-making model for benchmarking the healthcare costs for diverse treatment strategies for BRCA-mutated women with breast cancer. In particular, in our model, the costs savings are higher when all breast cancer patients undergo counseling and genetic testing before deciding on any diagnostic-therapeutic path, with a probability of obtaining savings of over 75%. This confirms the robustness of the decision-making model, which will provide increasingly beneficial results as its use increases (being applied to a larger population). Additionally, in this scenario, the path where the genetic test is performed before making any decision regardless of the therapeutic diagnostic chance (intensive follow-up radiological or prophylactic surgery) was proven to be the most advantageous. These findings are in line with results reported in some previous studies, although we provide some new insights. Our findings confirm previous research supporting the practice of extending the test to the population. A cost analysis study [54] for the English genetics service reported that the British NICE family breast cancer guidelines showed that testing all subjects with and without cancer but with a risk less than 5% was economically advantageous in women under the age of 59 years. However, whereas the implementation of the BRCA test appears cost-effective in ovarian cancer patients in England [45], studies demonstrating the cost advantage of a widespread test compared to the actual clinical practice are lacking. Additionally, there are no models of health economics that highlight any advantages of the pre-surgery test. In this sense, our study enriches the existing literature by offering a cost decision-making model that assesses the convenience of the pre-surgery test.

Comparative analysis between the two paths (intensive radiological follow-up and prophylactic surgery + ultrasound follow-up) shows a lower cost of the first, quantifiable, on average, as EUR 1,684 per single patient.

This type of decision-making, if applied systematically on a large scale, would lead to significant economic savings and an optimization of the resources that can be used for high genetic-familial-risk women.

As we considered the Italian case, we found that, according to AIOM data, in 2019, 936 new cases of BRCA-mutated patients were diagnosed among breast cancer patients, 795 new cases among ovarian cancer patients, and 675 new cases among pancreatic cancer patients for a total of 2,406 new diagnoses. Considering the increasing trend of these patients opting for bilateral prophylactic surgery in the presence of a positive test (about 80% in those affected by breast cancer according to the historical data of our Institute), it is clear that the extension of the use of the cost decision-making model throughout the national territory would result in significant savings.

The cost analysis upon which the decision-making model was built has much greater importance in the investigated setting where efficacy data are controversial, such as long-term survival in the various clinical paths. In the context of BRCA-mutated subjects, the pathway with the addition of periodic MRI leads to a higher number of early T1N0 diagnoses (56.3% vs. 29.2%), in diagnosis-positive lymph nodes (11.5% vs. 48.5%), and to a lower number of chemotherapies administered (47.9% vs. 80.2%) [55]. However, despite these data, the real impact of MRI on mortality reduction is still uncertain, particularly in BRCA2 carriers [9]. The choice to follow one path of treatment over another depends on having received a first diagnosis of cancer: In a recent study [56] conducted on 455 asymptomatic BRCA-mutated women who were not operated on, a clear preference for intensive radiological follow-up emerged compared to prophylactic mastectomy (68.8% vs. 31.2%). The increased acceptability of one path of treatment compared to another may also be affected by technological advances that allow increasingly better performance in the radiology field, as has been occurring in recent years in the field of automated image analysis and radiomics in the breast [57,58,59,60].

### Limitations and Further Research

This study was conducted by only analyzing the costs associated with the treatment strategy, thus adopting a national healthcare system perspective. Although an optimal cost decision is made on the premise of the cost plan to realize the goal of increasing economic efficiency, beyond the cost, it is important to consider the benefits to patients. Women with BRCA mutations need to make important choices regarding the appropriate therapeutic option at different times in their lives, whereas clinicians and health policy planners need to know the most effective and cost-effective risk-management options. A more complete and comprehensive analysis may be performed by benchmarking therapeutic paths considering the perspectives of other stakeholders. In future research, the model may include the patient standpoint, assessing the benefits of various therapeutic pathways for patients throughout their life. Additionally, the model provides support when choosing among diverse treatment strategies for BRCA-mutated women with breast cancer by benchmarking the healthcare costs. An important aspect that can be considered and included in the model is the preference of the patient towards a specific treatment, which can be the object of further research. 

## Figures and Tables

**Table 1:** Model input parameters: Probabilities.

Variable	Distribution		Source
Starting age (affected)	Normal	Mean = 40 SD = 2.5	[4,45,46]
Probability of being BRCA-mutation-positive in affected individuals	Uniform	Min = 10% Max = 20%	[4,47,48,49]
Annual risk of new incidence of breast cancer if BRCA-positive	20–29 years	0.005	[4]
	30–39	0.015	
	40–49	0.03	
	50–59	0.026	
	60–69	0.012	
	70–79	0.012	
Annual risk of contralateral breast cancer if BRCA-positive	20–29 years	0	[4]
	30–39	0.05	
	40–49	0.04	
	50–59	0.03	
	60–69	0.03	
	70–79	0.03	
Probability that patient is treated with radiotherapy after mastectomy		40%	Historical data
Probability that patient is treated with radiotherapy after quadrantectomy		95%	Historical data
Probability of undergoing genetic counseling	Bernoulli	45%	Historical data
Probability of undergoing BRCA genetic testing	Bernoulli	45%	Historical data
Probability of detecting suspected local recurrence (skin or lymph node recurrences)	Bernoulli	5%	Historical data
Risk of surgery complications	Uniform	min = 10% max = 20%	Historical data
Positive biopsy rate	Bernoulli	60%	Historical data

**Table 2:** Model input parameters: Probability of each therapeutic choice in the “as is” scenario.

Therapeutic Options	Data Inputs
% affected patients undergoing surgery after receiving BRCA test results (Path 30)	26%
% affected patients undergoing quadrantectomy before receiving BRCA test results (Path 10)	70%
% affected patients, BRCA-positive, choosing intensive breast screening (intensive follow-up) after quadrantectomy (Path 11)	20%
% affected BRCA-positive patients choosing bilateral mastectomy (RRM) and ultrasound follow-up after quadrantectomy (Path 12)	80%
% affected patients undergoing mastectomy before receiving BRCA test results (Path 20)	30%
% affected BRCA-positive patients choosing intensive breast screening (intensive follow-up) after mastectomy (Path 21)	20%
% affected BRCA-positive patients choosing contralateral mastectomy (RRM) and ultrasound follow-up after mastectomy (Path 22)	80%
% affected patients undergoing monolateral mastectomy after receiving BRCA test results, if BRCA-positive (Path 31)	70%
% affected patients undergoing bilateral mastectomy after receiving BRCA test results, if BRCA-positive (Path 32)	30%

**Table 3:** Model input parameters: Costs.

Activity	Cost (EUR)	Notes	Reference
Quadrantectomy	2,354	Without complications	NHS: DRG code 259
	2,717	With complications	NHS: DRG code 260
Intensive breast screening (intensive follow-up)	263.31	mammography and breast magnetic resonance imaging (MRI)	NHS: DRG codes 87371-88929-897
Biopsy	52.08	core-biopsy	NHS: DRG code 85111
Mastectomy including reconstructive surgery	8,265	Without complications	NHS: DRG codes 258-461
	8,872	With complications	NHS: DRG codes 257-461
Bilateral mastectomy including reconstructive surgery	16,530	Without complications	NHS: DRG codes 258-461
	17,744	With complications	NHS: DRG codes 257-461
Ultrasound follow-up	56.55	Breast examination and ultrasound	NHS: DRG codes 88731-897
Surgery for local recurrences (skin or lymph node recurrences)	4,583		NHS: DRG code 19881
Plastic surgery after complications or for breast implant replacement after 15 years	4,924		NHS: DRG code 461
Radiotherapy	2,936	cost per regimen in combination with systemic therapy	NHS: DRG code 409
Genetic counseling	20.01		NHS: DRG code 897B1
BRCA testing	1,107		Primary data collection

**Table 4:** Sensitivity and scenario analysis results.

		Net Unit Savings (EUR)			Optimal Therapeutic Path			
**Scenario 1**	**(A)**	Mean	2,982		Prob(Path 0)			55.00%
		SD	5,564		Prob(Path 11)			3.40%
		Min	0		Prob(Path 21)			1.60%
		Max	36,398		Prob(Path 30)			38.70%
		Prob(Net unit savings > 0)	34.20%		Prob(Path 31)			1.30%
**Scenario 2**	**(B)**	Mean	6,360		Prob(Path 11)			8.70%
		SD	6,458		Prob(Path 21)			4.20%
		Min	0		Prob(Path 30)			84.40%
		Max	39,000		Prob(Path 31)			2.70%
		Prob(Net unit savings > 0)	75.70%					
			**Net unit savings (EUR)**					
		**Starting age (years)**	**20**	**30**	**40**	**50**	**60**	**70**
	**(C)**	Mean	6,526	6,623	6,360	6,719	6,583	6,447
		SD	6,404	6,755	6,458	6,968	6,603	6,115
		Min	0	0	0	0	0	0
		Max	36,669	45,561	39,000	37,923	37,308	40,083
		Prob(Net unit savings > 0)	76.90%	76.20%	75.70%	75.70%	76.10%	76.60%
**Costs throughout the life of the patient of the most probable “as is” scenarios in BRCA-mutated patients (values in thousands (EUR))**								
		**Cost of Path 21**			**Cost of Path 32**			
**Scenario 3**	**(D)**	Mean	3,034		4,718			
		SD	7,329		11,199			
		Min	0		0			
		Max	32,204		44,105			

## Data Availability

The data presented in this study are available on request from the corresponding author. The data are not publicly available since they are the propriety of Istituto Tumori ‘Giovanni Paolo II’—Bari, Italy.
